# Experiences of HIV-related stigma among HIV-positive older persons in Uganda – a mixed methods analysis

**DOI:** 10.1080/17290376.2014.938103

**Published:** 2014-07-23

**Authors:** Monica O. Kuteesa, Stuart Wright, Janet Seeley, Joseph Mugisha, Eugene Kinyanda, Frederick Kakembo, Richard Mwesigwa, Francis Scholten

**Affiliations:** ^a^ MD, MPH, is affiliated to MRC/UVRI Uganda Research Unit on AIDS, Entebbe, Uganda; ^b^ is an Alumnus at BIARI, Providence, RI, USA; ^c^ MA, is a Doctoral Student at the School of East Asian Studies, University of Sheffield, Sheffield, UK; ^d^ PhD, is affiliated to MRC/UVRI Uganda Research Unit on AIDS, Entebbe, Uganda; ^e^ PhD, is affiliated to MRC/UVRI Uganda Research Unit on AIDS, Entebbe, Uganda; ^f^ PhD, is a Professor at the Department of Global Health and Development, London School of Hygiene and Tropical Medicine, London, UK; ^g^ PhD, is affiliated to MRC/UVRI Uganda Research Unit on AIDS, Entebbe, Uganda; ^h^ PhD, is an Alumnus at BIARI, Providence, RI, USA; ^i^ is an Associate Dean in the Department of Research and Post Graduate Studies, Uganda Christian University, Mukono, Uganda; ^j^ is an alumnus to BIARI, Providence, RI, USA; ^k^ is a Project Leader for the Expanded Kibaale, Kiboga Program at Infectious Disease Institute, Kampala, Uganda; ^l^ MSc, is affiliated to MRC/UVRI Uganda Research Unit on AIDS, Entebbe, Uganda

**Keywords:** disclosure, discrimination, HIV, older people, divulgation, discrimination, VIH, personnes âgées

## Abstract

There is limited data on stigma among older HIV-infected adults in sub-Saharan Africa. We describe the experiences of stigma and disclosure in a cohort of HIV-positive older people in Uganda. Using data from the Wellbeing of Older Peoples' Study of Kalungu (rural site) and Wakiso district (peri-urban site) residents, we measured self-reported stigma levels for 183 respondents (94 on antiretroviral therapy (ART); 88, not on ART) using a stigma score generated using three questions on stigma perceptions where 0 meant no stigma at all and 100 was maximum stigma. Based on two questions on disclosure, an overall score was computed. High disclosure was assigned to those who often or very often disclosed to the family and were never or seldom afraid to disclose elsewhere. We examined the experiences of HIV stigma of 25 adults (52% females) using semi-structured, open-ended interviews and monthly oral diaries over one year. Mean age of the respondents was 70 years (range 60–80 years) and 80% of all respondents were enrolled in ART. Interview transcripts were analysed using thematic content analysis. Overall, 55% of respondents had a high disclosure score, meaning they disclosed easily, and 47% had a high stigma score. The stigma scores were similar among those with high and low disclosure scores. In multivariate analyses with disclosure and stigma scores as dependent variables none of the respondents' characteristics had a significant effect at the 5% level. Qualitative data revealed that stigma ranges from: (1) perceptions (relatively passive, but leading to behaviour such as gossip, especially if not intended maliciously); to (2) discriminatory behaviour (active or enacted stigma; from malicious gossip to outright discrimination). Despite the relatively high levels of disclosure, older people suffer from high levels of stigma of various forms apart from HIV-related stigma. Efforts to assess for different forms of stigma at an individual level deserve greater attention from service providers and researchers, and must be context specific.

## Résumé

Les données sont limitées quant à la stigmatisation chez les adultes infectés par le VIH en Afrique sub-saharienne. Nous tentons ici de présenter les expériences de la stigmatisation et la divulgation chez une cohorte des personnes âgées séropositives en Ouganda. A l'aide des données tirées d'une étude sur le bien-être des personnes âgées menée auprès des habitants de Kalungu (site en milieu rural) et de Wakiso (site en milieu périurbain), nous avons mesuré les niveaux de stigmatisation déclarés par les 183 répondants eux-mêmes (dont 94 étaient sous les traitements antirétroviraux; 88 pas sous les traitements antirétroviraux). Les résultats étaient obtenus à partir de trois questions sur les perceptions de stigmatisation et en utilisant une échelle de 0 à 100, où 0 signifie aucune stigmatisation et 100, une stigmatisation maximale. A la base de deux questions sur la divulgation, on a fait des calculs pour obtenir une note globale. Une note élevée pour la divulgation était attribuée à ceux qui annonçaient souvent ou très souvent leur séropositivité à leurs familles et n'avaient jamais peur ou n'avaient guère peur de divulguer cette information par ailleurs. On a examiné les expériences de la stigmatisation liée au VIH chez 25 adultes (dont 52% du sexe féminin) au moyen d'entrevues semi-structurées et à questions ouvertes ainsi que les rapports oraux portant sur les expériences personnelles d'une année. Les répondants avaient un âge moyen de 70 ans (la gamme était de 60–80 ans). 80% de ces répondants étaient placés sous traitement antirétroviral (ARV). Les transcriptions des entrevues ont été analysées à partir d'une analyse thématique. Dans l'ensemble, 55% des répondants ont obtenu une note élevée pour la divulgation, c'est-à-dire qu'ils ont divulgué facilement leur séropositivité à d'autres, tandis que 47% ont obtenu une note élevé de stigmatisation. Les notes au niveau de stigmatisation étaient semblables chez les répondants ayant les notes élevées et chez ceux ayant les notes peu élevées pour la divulgation. Au niveau des analyses multi-variées, avec les notes pour la divulgation et pour la stigmatisation comme variables dépendantes, aucune des caractéristiques des répondants n'a eu d'effet significatif au seuil de 5%. Les données qualitatives ont indiqué que la stigmatisation varie entre : (1) les perceptions (relativement passives mais entraînant les comportements tels que les commérages notamment s'ils ne sont pas mal intentionnées); et (2) les comportements discriminatoires (stigmatisation active ou effective; allant des médisances à la discrimination pure). Malgré les niveaux relativement élevés de divulgation, les personnes âgées souffrent des niveaux élevés de stigmatisation se manifestant sous formes variées outre la stigmatisation liée au VIH. Les efforts pour évaluer les formes différentes de stigmatisation au niveau individuel méritent une plus grande attention de la part des prestataires de services et des chercheurs. Cette communication met en exergue la nécessité de déployer les efforts qui sont adaptés à chaque contexte culturel spécifique dans le but d'atténuer la stigmatisation chez les personnes âgées maintenant et dans l'avenir.

## Introduction

HIV remains a highly stigmatised condition worldwide (Brennan & Karpiak 2010; Genberg, Hlavka, Konda, Mamanc, Chingono, Mbwambo, *et al.* 2009; Seeley, Zalwango, Mugisha, Kinyanda, Wake & Scholten 2010). The fear of stigma negatively affects the way in which individuals and families protect themselves, seek care and treatment and provide support to those affected with HIV, which may increase their susceptibility and vulnerability to HIV infection (Genberg *et al*. 2009; Pharris, Phuong Hoa, Tishelman, Marrone, Kim, Brugha, *et al*. 2011; Turan, Miller, Bukusi, Sande & Cohen 2008; Uganda Ministry of Health, ICF International, CDC, USAID & WHO 2012). Older people (50 years and older) living with HIV may face stigma because of both age and their HIV status (Emlet 2006b); yet there is a paucity of information to inform interventions to address stigma among older people, and to measure or monitor the progress of those interventions (Nyblade 2006).

The number of HIV-positive older persons worldwide is unknown but likely to be increasing because of improved access to antiretroviral therapy (ART) (High, Brennan-Ing, Clifford, Cohen, Currier, Deeks, *et al*. 2012; Negin & Cumming 2010; UNAIDS 2013), which results in longer survival. There is also evidence that some members of this age group are acquiring the infection in later life. However, the magnitude of these phenomena is yet to be quantified (Hontelez, Lurie, Newell, Bakker, Tanser, Barnighausen, *et al*. 2011; Negin & Cumming 2010; Seeley *et al*. 2010; UNAIDS 2013; WHO, UNAIDS & UNICEF 2012). In addition to gathering information on HIV among older people it is critical to consider the role of HIV- and AIDS-related stigma in the daily lives of older people living with HIV. This information can inform the design and implementation of effective prevention and treatment programmes which address the needs of older people living with HIV.

The population of older Ugandans is projected to increase from 1.379 million (3.9%) of the total population in 2012 to 5.4 million, by 2050 (UNDP 2008, 2011). Therefore, the number of older people is likely to increase. There is an increasing number of older people infected with HIV which is likely to impact on the care needs of older people with HIV in Uganda. The HIV prevalence among older Ugandans aged 50–54 is 7.1%, and 5.7% for the 55–59 age group, compared to 6.7% in the 15–49 age group (Uganda Ministry of Health *et al*. 2012).

HIV stigma has been defined as ‘prejudice, discounting, discrediting and discrimination directed to people perceived to have AIDS or HIV, and the individuals, groups, and communities with which they are associated’ (Herek 1999). There are at least four ‘domains’ of HIV-related stigma and discrimination: (1) the fear of HIV transmission through casual contact with people living with HIV, (2) negative judgments/beliefs about people living with HIV, (3) ‘enacted’ stigma or experiences of discrimination related to AIDS, (4) layered stigma, where HIV-related stigma combines with stigma towards otherwise excluded or marginalised groups (Obermeyer, Bott, Carrieri, Parsons, Pulerwitz, Rutenberg, *et al*. 2009). Stigmatising beliefs are the product of multiple social influences including attributions of responsibility for being different from the majority as well as beliefs that individuals are contaminated and tainted (Goffman 1963). People living with HIV face stigma and discrimination in a variety of contexts, including the household, community, workplace and health-care settings (Kuteesa, Seeley, Cumming & Negin 2012). Recent reports show that HIV-related stigma remains a significant problem across vulnerable groups such as women, older adults, sexual and ethnic minorities, because many people still hold beliefs that may stigmatise or lead to discrimination against these individuals (Emlet 2007; Kaiser, Family & Foundation 2009; Murphy, Austin & Greenwell 2006).

According to Deacon and others, the combined effect of symbolic stigma (based on moral judgement, for instance, when links are drawn between the disease and promiscuity) and instrumental stigma (based on biological factors such as severity, contagiousness and treatability) makes HIV more stigmatised than diseases such as cancers and hepatitis. Furthermore, either symbolic or instrumental stigma may result in discrimination (Deacon, Stephney & Prosalendis 2005). It is worth noting that all negative beliefs and behaviours against people living with HIV are not necessarily caused by HIV stigma.

Studies examining HIV-related stigma across the life span are few. Studies highlighting the varied manifestations of stigma or its impact on the lives and general wellbeing of older adults are even fewer. Evidence shows that the magnitude, experience and perceptions (e.g. discrimination, prejudice, violence, etc.) of HIV/ AIDS-related stigma, its determinants and negative sequelae differ across age, cultures and environments (Kaiser & Miller 2001). Generally, older adults experience greater overall and perceived stigma and tend to disclose HIV status to fewer individuals than their younger counterparts (Emlet 2006a).

Substantial research on HIV-related stigma has been done in settings with concentrated epidemics where layered stigma due to multiple, interrelated stigmatising attitudes such as intravenous drug use and gay or bisexual orientation exists, which mutually reinforce each other. These interrelated stigmas often result in further marginalisation of vulnerable groups partly because they predict negative behavioural intentions (Genberg *et al*. 2009; Herek 1990; Nyblade 2006; Pharris *et al*. 2011). These studies have documented various, social, psychological and physical challenges associated with older HIV-positive adults compared to their younger HIV-positive counterparts. These include inadequate support networks, isolation loneliness and depression and suicidal ideation (Emlet 2006c; Emlet, Tozay & Raveis 2010; Karpiak, Shippy & Cantor 2006). Other psychosocial issues include stigma and discrimination, and concerns for disclosure (Emlet 2006a, 2008; Grov, Golub, Parsons, Brennan & Karpiak 2010). However, less is known about the features of HIV-related stigma in generalised epidemics. This information is needed because HIV-related stigma may still be heightened in sub-populations of older persons who are vulnerable to the sociocultural and socio-economic processes that shape the social inequalities, fuel HIV epidemics and influence access to and utilisation of prevention, treatment and care services (Castro & Farmer 2005; Genberg *et al*. 2009).

Using both qualitative and quantitative data from Uganda, this paper presents data on the measurement of stigma and disclosure among older HIV-positive persons and, using longitudinal qualitative data, investigates the different and changing manifestation of HIV-related stigma among older people. The paper also describes and explores other forms of stigma which older adults may experience related to ageing and poverty and how these experiences affect their lives and wellbeing; with a view to inform efforts to reduce stigma in this sub-population.

## Methods

### The setting and study population

This study was conducted in 2009–2010 by the Medical Research Council/Uganda Virus Research Institute (MRC/UVRI) in collaboration with the World Health Organization; in a rural site in Kalungu district located in south-western Uganda and in a peri-urban site in Wakiso district, located in and around Entebbe town near Kampala, the capital city of Uganda, on the shores of lake Victoria. The MRC/UVRI, Uganda Research Unit on AIDS has been conducting HIV/AIDS-related research in these two sites since 1989.

The rural site recruited mainly within the General Population Cohort (GPC) located in Kalungu district in rural south-western Uganda. A few participants were recruited from the AIDS Support Organisation (TASO), an organisation that cares for people with HIV in Uganda.

The GPC was established by the MRC/UVRI, Uganda Research Unit on AIDS in 1989 to study the epidemiology of HIV (Asiki, Murphy, Nakiyingi-Miiro, Seeley, Nsubuga, Karabarinde *et al*. 2013). MRC/UVRI has conducted annual demographic and serological surveys in the GPC for the past 24 years. Data collected annually include demographic, socio-medical and serological data that are collected from all residents within the GPC. The main occupation for people living in the GPC study villages is subsistence farming. The majority (70%) of the population constitutes ethnical Baganda. However, 15% are immigrants from Rwanda; about 4% of the population constitutes immigrants from Tanzania. The rest of the population is a mixture of other tribes. The community's largest religious denomination is Roman Catholic (60%) with 17% Protestant and 23% Muslim. Over 10% of the population is aged 50 years and over. Socio-economically, most households own less than five acres of land; relatively, few households are landless.

The Entebbe cohort was established by the MRC/UVRI Uganda Unit on AIDS in collaboration with TASO Entebbe, and was an open cohort study that followed HIV-infected participants from 1994 to 2009. The aim of this cohort was to evaluate some interventions to reduce morbidity and mortality in people infected with HIV (French, Nakiyingi, Carpenter, Lugada, Watera, Moi *et al*. 2000). Many residents in the peri-urban site lived in rural areas around Entebbe and were not residents of the town (‘peri-urban’ in this context refers to the site of service provision, not place of residence). At the peri-urban site, some of the study participants were recruited from those who had participated in the Entebbe cohort as well as some participants from TASO in Entebbe. The population in Wakiso district is more mixed than that in Kalungu, because people from many different tribes have settled near Entebbe. Several Christian denominations as well as a few Muslim groups are represented; however there is a strong Roman Catholic presence. Livelihood in the rural areas around the town is mainly dependant on cultivation, fishing and various forms of trade. A few people are involved in formal employment in fields of teaching, health care and cleaning services. ART was introduced in both study areas in early 2000 and drugs are available free of charge. ART coverage rates are high because HIV testing services are available at both sites. Older people with HIV receive treatment from ART clinics for adults in these two sites.

The Well Being of Older Peoples' Study (WOPS I) was part of the bigger quantitative study by WHO on Global Ageing and adult health. The study consisted of 510 HIV-positive and -negative people aged 50 years and older divided in five groups (256 in the rural area and 254 in the urban area, 61% women and 39% men). The mean age overall was 65 years, but for the HIV-positive groups (4 and 5) the mean age was 59. The five groups constituted older people who have an adult child who died of AIDS; have an adult child who is living with HIV and on ART; have no child with HIV/AIDS and are not infected with HIV themselves (comparison group); are HIV infected and on ART for at least one year; and are HIV infected and not on ART.

Each group comprised about 100 respondents with half of the respondents recruited from the rural site and half from the urban site. At both sites, a list of all older people was first drawn based on the surveillance field site census records and nested surveys. For each study group about 100 people were randomly selected from these study lists. Refusals were less than 1%.

## Data collection

### Interview process

#### Quantitative

Data on HIV-related stigma were collected using a structured questionnaire as part of a larger study on the wellbeing of older people. The questionnaire used was based on the SAGE (Study on global AGEing and adult health) and was pretested and piloted prior to use (Scholten, Seeley, Mugisha, Zalwango & Wright 2011b) The study attempted to measure HIV/AIDS stigma by asking HIV-positive respondents from two groups: those on ART and those not on ART. The tool included a series of questions about how they felt during the 12 months prior to the survey. The purpose of this was to assess the prevalence of stigma and to illustrate the influence of ART on perceptions of stigma perhaps leading to confidence and least affected wellbeing, as well as improved health which empowered the individual which was reflected in the way they were treated by others. Five stigma-related questions were put to HIV-positive respondents; the respondents were asked whether a specific stigma-related problem happened never, seldom, sometimes, often or very often. The questions concerned: (1) worry ‘that others will view me unfavourably because I am HIV+’; (2) being in situations ‘where others say offensive things about people with HIV’; (3) about having ever been ‘treated as less competent by others when they learn that I am HIV+’; (4) about disclosure of HIV status, and having ‘avoided telling others outside my immediate family that I am HIV positive’; and (5) disclosure to family members.

#### Qualitative

Interviews were conducted through the investigation of life histories and oral diaries, using a semi-structured extended conversation designed to provide qualitative insights into perceptions of stigma and the impact of stigma on the wellbeing of older persons. It was also aimed at providing a valuable understanding of the different forms of stigma and relationships between them.

The interviews also investigated participants' hopes, concerns, perceptions, expectations and experiences. The life stories and oral diaries were written up from notes by interviewers (tape recorders were not used due to concerns from some participants about recordings) immediately after each interview. The interviews were conducted in Luganda (known to both participant and interviewer) and were translated and transcribed into English.

All interviews were conducted by experienced interviewers (two men and five women, all but 1 aged above 60 years). Interviewers made monthly visits to each of the 40 respondents over a period of one year and collected a monthly oral diary. The diary focused specifically on the week prior to each interview. It was expected that this time span (the seven-day-recall period) was ample for respondents to recall the detailed experiences they had had overtime. For each respondent, an additional interview was conducted specifically to focus on documenting their life history.

#### Research ethics

Permission was sought and granted by the Science and Ethics Committee of the UVRI and the Uganda National Council for Science and Technology. Individuals who were included in the study gave written consent after the study purpose had been explained. Respondents requiring medical attention at the time of interviews were referred to the MRC/UVRI study clinic.

*Quantitative data analysis*: Data were cleaned and analysed using STATA 11 software. Based on the two questions on disclosure, an overall score was computed. High disclosure was provided to those who often or very often disclosed to the family and never or seldom were afraid to disclose elsewhere. All others received a low disclosure score. The responses on the three questions on stigma perceptions were combined into one stigma score where 0 meant no stigma at all and 100 was maximum stigma.

*Qualitative data analysis*: Data were managed using NVIVO 8 software and were analysed by thematic content analysis. During the primary data analysis, the study team discussed the emerging themes and sub-themes, both manual and electronic analysis have been employed during secondary data analysis.

## Results

### Quantitative findings

In total 510 older persons were interviewed in the quantitative component: 50% in the rural area and 50% in the peri-urban area, 61% women and 39% men; mean age overall was 65 years, but for the HIV-positive groups the mean age was 59. The findings of this study are reported elsewhere (MRC/UVRI 2011; Scholten, Mugisha, Seeley, Kinyanda, Nakubukwa, Kowal, *et al*. 2011a).

The quantitative analysis for this paper is based on all the HIV-positive respondents (*n* = 182), 94 of whom were on ART and 88 who were ART naïve from both sites who responded to the stigma questions (Table 1). About half of the participants were resident in a rural area and half in a peri-urban area.

**Table 1.** Background characteristics of respondents (*n* = 182), for quantitative assessment.

Characteristic	Group	No. of HIV on ART (%)	No. of HIV not on ART (%)	Total
		(*n* = 94)	(*n* = 88)	(*n* = 182)
Sex	Male	40 (42.6)	34 (38.6)	74
	Female	54 (57.5)	54 (61.4)	108
Age	50–59	53 (56.4)	55 (62.5)	108
	60–69	31 (33)	18 (20.5)	49
	70+	10 (10.6)	15 (17.1)	25
Residence	Rural	46 (49)	40 (45.5)	86
	Urban	48 (51.1)	48 (54.6)	96
Education level	None	14 (14.9)	21 (24.1)	35
	Primary	56 (59.6)	49 (56.3)	105
	Secondary	21 (22.3)	14 (16.1)	35
	Higher	3 (3.2)	3 (3.5)	6
Current marital status	Never married	1 (1.1)	1 (1.1)	2
	Married/cohabiting	23 (24.5)	27 (30.7)	50
	Divorced/separated	17 (18.1)	22 (25)	39
	Widowed	53 (56.4)	38 (43.2)	91
Permanent income quintile	lowest	17 (18.1)	21 (23.9)	38
	Second	17 (18.1)	21 (23.9)	38
	Middle	20 (21.3)	19 (21.6)	39
	Fourth	21 (22.3)	16 (18.2)	37
	Highest	19 (20.2)	11 (12.5)	30

Table 2 shows the responses to the specific questions. Overall, 46% said they very often disclose their HIV status to others, and an additional 20% said often. Only 14% said never or seldom. Also, two-thirds of respondents said they never avoided disclosure to others outside the family. On the other hand, 22% said they often or very often avoided such disclosure.

**Table 2.** Frequency distribution of responses to the five questions on disclosure and stigma among 182 HIV-positive respondents aged 50 and over.

	Never	Seldom	Sometimes	Often	Very often	Total
Disclosure of HIV-positive status to others	9.3	4.4	20.3	19.8	46.2	100.0
Avoid disclosure of HIV status outside family	66.0	4.3	7.5	10.6	11.7	100.0
Been in situations where others say unfavourable things about people with HIV	26.9	9.3	19.8	22.5	21.4	100.0
Worried about others viewing them unfavourably because I am HIV positive	59.3	11.0	8.2	8.2	13.2	100.0
Feels treated as less competent because of HIV positive	63.7	6.6	12.6	7.1	9.9	100.0

Nearly half of the respondents (44%) said they were often or very often in situations where people spoke unfavourably of people living with HIV. On the other hand, 36% said they had never or seldom been in such a situation. Most respondents (70%) were never or seldom worried about others viewing them unfavourably because of HIV, but 21% worried about it often or very often. About one in six respondents were (very) often worried about people treating them less favourably because they were HIV positive, but the majority never worried about this.

Table 3 shows the results on the disclosure and stigma scores by respondent characteristics. Overall, 55% of respondents had a high disclosure score, meaning they disclosed easily, and 47% had a high stigma score. The stigma scores were similar among those with high and low disclosure scores. Women had lower disclosure and perceived higher stigma than men, although the differences were not statistically significant at the 5% level. Similarly, participants on ART had higher disclosure rates than HIV positives who were not on ART (61% and 49%, respectively), but also had slightly higher stigma scores (49% and 44%, respectively). Rural–urban differences were small. The poorest 40% had lower disclosure and lower stigma than the top 60%. There were no significant differences by age, with 60–69 years having the poorest scores.

**Table 3.** Disclosure and stigma scores by respondent characteristics.

		Disclosure score		Stigma score	
	Number	%	SE	%	SE
All	182	54.9	3.7	46.7	1.8
Women	108	50.0	4.8	48.6	2.4
Men	74	62.2	5.7	43.8	2.9
On ART	94	60.6	5.1	49.0	2.6
Not on ART	88	48.9	5.4	44.2	2.6
Rural	86	52.3	5.4	47.4	2.7
Urban	96	57.3	5.1	46.0	2.5
Poorest 40%	76	53.9	5.8	43.7	2.7
Top 60%	106	55.7	4.8	48.8	2.5
No education	35	45.7	8.5	48.2	4.4
Primary	88	60.2	5.2	46.4	2.6
Secondary +	54	53.7	6.8	46.3	3.3
Disclosure high	82			47.4	2.7
Disclosure low	100			46.1	2.5

Note: SE, standard error.

In multivariate analyses with disclosure and stigma scores as dependent variables none of the respondents' characteristics had a significant effect at the 5% level.

### Qualitative findings

For the qualitative study, 40 participants (20 from each site) were selected from the quantitative study sample. These participants were aged 60 years and over (mean age = 65), in order to focus on people who may be more likely to face the challenges of failing health.

The qualitative analysis of this paper is based on 25 HIV-positive participants from both the rural and peri-urban sites (Table 4). Because there was a larger number of people aged over 60 who were HIV-positive in the peri-urban setting, a decision was taken to focus on this group in that sampling. Therefore, all 20 peri-urban participants (9 men, 11 women) were 60 years and over; HIV positive and receiving free care from TASO in Entebbe. Fourteen had been on ART for at least one year, while the remaining six were not on ART but were taking cotrimoxazole. Five rural participants (three men, two women) were HIV positive and receiving care from MRC/UVRI; four were on ART, one was ART naive.

**Table 4.** Background characteristics of respondents (*n* = 25) for qualitative assessment.

Sex	Male	12
	Female	13
Age	80>	1
	70–79	13
	60–69	11
Location	Kalungu	5
	Wakiso	20
ART	On ART	18
	Not on ART	7
HIV care centre	MRC/UVRI	5
	TASO	20
Tribe	Ethnic Baganda	13
	Rwandese/Burundian origin	7
	Others	5
Marital status	Married	7
	Widowed	12
	Separated/divorced	6
	Other^a^	0
Living	Lived alone	8
	Lived with grandchildren/other relatives	17

^a^Cohabiting, never married.

The qualitative research mainly focused on general circumstances and experiences obtained from the qualitative study throughout the year. The details are discussed in the section that follows. Data were generated from 25 HIV-positive participants from both the rural and peri-urban sites. The data depict feelings, perceptions and manifestations of stigma attributed to physical health problems (related to ageing as well as HIV). The data also reveal other factors that intensify stigma such as financial problems, witchcraft accusations and fears, caregiving responsibilities, isolation and loneliness, general mood, lack of support among others and death of a close relative. Collectively, experiences and perceptions related to these factors influenced participants' subjective explanations of the causes, perceptions and ratings of stigma.

#### Self-stigma

We found that the internalisation of stigma was often referred to as ‘self-stigma’. Some respondents discussed HIV testing and how they confronted the prospect of HIV/AIDS; they used the overarching term ‘stigma’ to describe what has been identified here as: (1) social stigma and (2) health fears. This is illustrated by a comment from Paul (male, age 59); who developed HIV/AIDS symptoms in the late 1990s and went for a test, not at the nearest centre where he reportedly ‘feared the stigma’ but at facility in Entebbe where he thought nobody would know him.

#### Poverty

Different perceptions about stigma in association with poverty were expressed by respondents. Stigma was also linked to worries about quality of life and survival, and also associated with anticipated loss of productivity due to deteriorating health. The fears expressed by some individual respondents were primarily concerned with survival of an individual and his or her dependants (i.e. worries about an inability to care for them). In such cases, there was less anxiety about the opinions, criticisms or actions of other people. For example, Vincent (male, age 65) worked as a fishmonger and engaged in private agricultural activities; as a result he seemed to be faring better economically than many other older HIV-positive respondents. While Vincent complained that money ‘ … will never be enough … ’ he had made many preparations in the hope that his family will survive economically in the event of his death. All his concerns were ‘pointing to poverty’ and the fact that HIV/AIDS was ‘becoming a stumbling block’. David (male, age 60) seemed conscious that both his poverty and HIV status were conspicuous, and this awareness seemed to affect his wellbeing. He referred to the people he met at the centre where he collected his medicine: those with money could easily be identified by their looks; they looked healthy and strong; their appearance did not show whether or not they were infected with HIV. David said that he would like to have an HIV-positive spouse so that they could settle down and he could get rid of loneliness.

It can be done but the financial aspect doesn't allow me to have a woman, that's why I postponed the idea to a later date when I will have money to restore my former status of health.

In many cases such as those described above, the sense of personal stigma was influenced by socio-economic factors. The feeling of stigma was reduced if an individual engaged in income-generating activities that kept them financially independent, busy and generally happy. This applied to males and females and to people of different ages.

#### Health fears

The second understanding of ‘stigma’ alluded to by a number of respondents was related to health fears including fear of deteriorating health and fear of dying. For example, Grace (female, age 73) reported that her first husband and one of her co-wives had died of an unknown disease; in her second marriage, one of her co-wives died of an unknown disease, and when the second husband died, people started saying that all his lovers had also died of AIDS. Grace first suspected that she had acquired the virus ‘some time back’ when she developed a skin disease that ‘failed all the doctors’. She then encountered further social stigma when relatives and friends ‘feared to associate with her so the stigma increased’. Grace ‘feared having got AIDS’ as if more a reaction to, or internalisation of, social stigma than to the prospect of HIV/AIDS in health terms. However, she said that she then ‘withstood all the fear’, and took an HIV test. On testing HIV positive, ‘the fear intensified’ and the health (HIV/AIDS) fear became a reality. Faith (female, age 70) had encountered stigma in the past: she had taken care of her nephew who had been neglected by relatives when diagnosed HIV-positive. She also reported that her son supported her to face the prospect of being HIV-positive. He took her for HIV testing and also helped her to overcome social stigma by enabling her to confront HIV/AIDS as a health issue. He helped to close the gap that had been created when other relatives abandoned her. She continued to say:

Without my son's support, I would probably be dead by now because I was dying of stigma and misery in the house.

Respondents frequently described how access to drugs and counselling made a difference to their lives and reduced self-stigma. ART in particular is often described as providing renewed physical energy and improved health as well as hope that HIV/AIDS is an illness that can be endured while living a normal life. Furthermore, respondents on ART had increased access to health services and counselling from providers such as TASO, which incorporated advice and appropriate messages regarding stigma. Unsurprisingly, participants usually reported that after receiving ART services from TASO, they learnt to deal with stigma and were more open about their HIV status.

A case in point is a 63-year-old female respondent named Patricia who perceived HIV/AIDS in health terms and seemed to have no anxiety about social stigma. She had suggested to her husband that they both go for HIV testing but the husband refused, despite the fact that his condition was deteriorating. Her husband and their son died of AIDS in 2001. After developing rashes on her legs, Patricia became proactive and went for a test in 2002. She said that after testing HIV-positive, she disclosed to her family members and church leaders in the Catholic and Anglican Church of Uganda. Probably because of this, she says that she never experienced any problems with family or neighbours and their children. With regard to the respect and care given to her, Patricia attributes it to her former work (she was once a local chairperson for women's affairs) she said that she ‘cannot be easily forgotten’ and besides they ‘keep on following her for advice’ and she was treated locally as a grandparent (implying respectfully) and has many visitors.

Patricia notes that other people in a similar condition were not as self-assured. She observed that: *AIDS is a disease people should not fear because it is like suffering from malaria*.

During one interview, she reported having been to an engagement party where she saw that some of her friends were also sick, but they ‘feared to interact with others’. This fear, Patricia said, ‘is why people are dying without getting help from TASO and other places’. However, despite her confidence, Patricia recounted that at the same event a stepdaughter greeted Patricia and asked why her face was ‘having rashes’ on her face. Patricia replied that she had been bitten by mosquitoes because her ‘mosquito net was washed and not replaced immediately’. Perhaps the stepdaughter was unaware of Patricia's HIV status or perhaps the respondent did not wish to dwell on her health on that occasion and brushed the issue aside. Despite her openness and pragmatic viewpoint, Patricia was still secretive and sensitive to stigma.

#### Constant reminders

Respondents indicated that several things reminded them of HIV-related stigma even when they thought that they were no longer concerned about it. Skin changes such as black spots and skin rashes, general body weakness, poverty and loneliness served as reminders of their status. Joyce (aged 60) who was not yet on ART (because her CD4+ cell count was high) but on Cotrimoxazole, reported physical pains ‘constantly attacking’ her, preventing enjoyment of life and causing insomnia. Joyce bleakly explained that she thinks a great deal about the ‘deadly disease, which is never going to cure’ and she was worried that she may die anytime. Diana (aged 70) pointed out that attending social gatherings always made her feel out of place because such gatherings made her think of her condition.

#### Pre-emptive coping strategies

The anticipation of discriminatory behaviour could lead to pre-emptive strategies to avoid disclosure and discrimination, even in cases where the HIV-infected individual did not subscribe to those stigmatising beliefs. This condition may be viewed as an indirect form of internalisation of stigma. Some respondents expressed denial as a means of coping with stigma. Naki (a woman aged 73): *I have no stigma even if I attended a function I wouldn't care because the whole world is infected with HIV*.

Another respondent Dorotiya (a woman aged 70) reported that the reason she did not associate much with village members was because they gossiped a lot. However, she apparently felt less stigmatised now than before and was serving as a ‘consultant’ in the village for those who are interested in learning about HIV and AIDS. Nonetheless, she admitted to occasionally attempting to hide her HIV status because of the fear of being scorned by those who did not know.

Other forms of coping strategies included change of identity and address when accessing healthcare to avoid being stigmatised. Two respondents preferred to travel longer distances rather than access care locally for fear that they could easily be identified by neighbours or, for some, by their sexual partners. Dorotiya (mentioned above) received the first round of ART treatment from TASO. She then opted to register at another health centre under a different identity. Later, she made arrangements with TASO to deliver medication to her home. Another example is Felista (female, age 64), who had concealed her maiden name, preferring instead to be identified by her husband's name at home. She used his name in daily life with friends but used her maiden name when accessing HIV treatment. She was concerned that otherwise, ‘people will talk about her status’ and laugh and gossip about her because she was HIV-positive. However, others had a different experience with their own neighbours and friends: Teddy (female, age 66), for example, acknowledged that although stigma still existed in her community, her neighbours were very supportive:

[ … ]am used to the disease and am ready to move on with life … my relationship with my neighbours is good and there is no stigma … no one pin points at me, or laughs at me … this gives me courage to continue taking my ARTs and septrin [ … ] maybe God will come to my rescue and heal me.

#### Age-related stigma

HIV/AIDS stigma may be less of a problem for older people than age-related stigma. Many participants commented that stigma intensified in situations where aged people lived alone and had no carers. Linda said she maintains a good relationship with family, relatives and the community, which is why people giver her food and sometimes even money. The neighbours' two small children visit her daily and ‘keep me busy so that I do not feel lonely’. However, she reported that people laugh at her when they see her digging because in *Buganda*, an older and sick person ‘is not supposed to dig’. However, despite this criticism Linda expressed pride to the interviewer in still being able to do things herself, rather than begging.

Among the Baganda, like some other African cultures, older people particularly elderly women are often suspected of using witchcraft or charms but also of being affected by such supernatural powers. This has implications for their access to healthcare. Furthermore, death or a disease may be interpreted in the idiom of witchcraft. A disease or other cause of death may not necessarily be considered a true or independent cause of death (Federici 2012; Livingston 2003). A case in point is David (age 60) who reported that people told him a witch had cast a spell on him when he first became sick with HIV/AIDS. In 2006, he began ‘feeling fever, then herpes zoster (*Kisipi*)’. His friends convinced him that he was ‘charmed’; and that the sickness brought about by magic sent to torture him. David sold all his property to pay to visit traditional healers. The first healer told him that the woman he had neglected was responsible for sending him these ‘charms’ that made his body sick. When the herbal medicine he was given by the first healer did not help, his landlady and a few other friends then advised him ‘to abandon witchcraft’. By this time, David was at an advanced stage of HIV/AIDS; he could not cook for himself so the landlady ‘felt compassion’ and began looking after him.

The belief in witchcraft occasionally affected people in a different way. Some people in the community tended to think that charms were used by some infected persons to persuade people to visit them. Joyce (a woman in her 60s) said that a neighbour accused her of influencing visitors to come and visit her only (and to ignore other sick persons). This might have just been the result of envy from one neighbour – an exception to Joyce's otherwise good relationship with the community. During two interviews, three months apart, Joyce expressed concern about this neighbour who was ‘very violent’ towards her ‘in words only’. The neighbour would block the way of visitors to Joyce's house and claim that:

The pastor always goes to her home; she cannot pass a day without getting visitors’, and … cars come from Entebbe but they only visit her. She has charms that influence these visitors to come only to herself.

Joyce said this had tarnished her name in the whole community. While this may be a case of enacted stigma or discrimination it may be more resentment at the attention Joyce was getting rather than specifically as HIV/AIDS discrimination.

#### Changes over time

The qualitative data, collected over the course of the year, showed that the experience of stigma was not constant. The examples above show that stigma may be felt in some situations and not in others, and it varies over time. Grace (Table 5), for example, said that relatives and friends had earlier ‘feared to associate with her’ when she developed a skin disease. However, she said this fear decreased over time. She illustrated this by referring to three daughters of her neighbours who now enjoyed resting in the shade beside her home despite the fact that they know her to be a registered client at TASO.

**Table 5.** A summary of stigma-related comments extracted from Grace's (female, 73) oral monthly diary.

Month	Attitude to HIV/AIDS and stigma
1	Grace said that people with HIV/AIDS should not fear to join TASO ‘to help them kill the stigma’
7	She said she feels out of place at social gatherings (parties, weddings, religious events) because she thinks of her condition ‘every now and then’. However, this is perhaps more of a preoccupying health anxiety than HIV/AIDS-related self-stigma
10	She described herself as a very social person, known and treated well in her community. She proudly said that this is why the neighbours directed the interviewer to her that day without hesitation. When she attends ceremonies (i.e. funerals) she asks for food and meat to take home; she no longer feels ashamed to carry food home. However, In one of the interview transcripts, the interviewer commented that Grace is a ‘cruel old lady whose moods change from time to time, so counselling has been on and off to maintain our discussion flow’
11	She said that unless there is a problem or a function to attend, she does not associate much with village members because they gossip a lot. The interviewer commented that the respondent ‘fears being laughed at’. The subject and tone of the anticipated gossip were not stated in the interview transcript, the interviewer noted that Grace ‘fears being laughed at’ although, the interviewer added, there is ‘less stigma now than there used to be’. The respondent proudly declared that she is an HIV/AIDS ‘consultant’ in her village; she advises people to go to TASO for HIV tests. However, gossip does not necessarily have malicious intent; it is not necessarily discrimination or enacted stigma, and a wish to avoid gossip is not necessarily driven by fear
12	Grace was more defiant with regards to gossip, and typically defiant with regards to stigma. Grace said she ‘has no stigma’ and that even if she went to a function she would ‘not care’ because ‘the whole world is infected with HIV/AIDS’. During the twelfth monthly interview, commenting that she ‘has no stigma’ and that even if she went to a function she would ‘not care’ because ‘the whole world is infected with HIV/AIDS’. In this way, Grace seemingly takes a determined attitude towards stigma, seeing it as something that should be fought against, that her HIV status presents a choice of acceptance or denial: that although HIV/AIDS is prevalent, stigma does not need to be
	This confidence stretches beyond HIV/AIDS-related stigma, perhaps to encompass poverty-related stigma: Grace said her appetite is good and when she attends ceremonies (i.e. funerals) she eats a lot and asks for food and meat to take home. Whereas she used to feel ashamed to carry food home, now she does not mind

## Discussion

In keeping with other studies on HIV and older people, our findings suggest that older persons experience a considerable amount of internalised and enacted stigma (Emlet 2006b, 2007). First, relatively high levels of stigma were identified in the quantitative survey. However, it was also notable that in this rural and peri-urban population of older people in Uganda a considerable proportion of respondents disclosed their status within and outside the family, with about two-thirds disclosing their status often or very often and two-thirds never or seldom avoiding disclosure outside their family.

Second, the availability of ART may have a destigmatising effect in these communities. Indeed, respondents on ART more often disclosed their status than others and also had low stigma scores, although the difference was not significant. But there may also be an indirect effect of ART availability on lowering stigma and enhancing disclosure among those who have not yet initiated treatment. It is also worth noting that being identified as ‘different’ by society might in itself be a source of stigma because it labels one as ‘different’ (Goffman 1963). Goffman's description of the social label of difference which compels stigmatised individuals to view themselves and others to view the stigmatised as different emphasises the fact that stigma arising from this form of ‘othering’ could complicate the uptake of HIV care and treatment even in situations where individuals are asymptomatic.

The lack of major differences in stigma and disclosure by respondent characteristics such as place of residence, age and socio-economic status was also notable, although women had somewhat less disclosure and more stigma than men.

Elsewhere, previous literature suggests that older age is associated with increased HIV stigma and less disclosure of HIV status (Emlet 2006a). The findings in our study could partly be due to reporting bias, or to the project influence, or to other issues. In particular, the existence of HIV research projects may have positive effects on disclosure and stigma.

It is worth noting that the accounts in our study do not necessarily represent a general trend with regard to gradual reduction of stigma. They are apt illustrations which partly represent subjective viewpoints influenced by many factors that have significantly contributed to this optimism. These factors include the high prevalence of HIV/AIDS which might lead to its increased acceptance of older adults' HIV status, the availability and effectiveness of counselling as well as ART to suppress the HIV virus and improve health outcomes. However, HIV stigma remains high among underserved populations and particularly has implications for older adults in terms of; (1) access and utilisation of HIV testing and treatment services (2); physical and mental health and wellbeing, and (3) access to social support (Letamo 2003; Wen, Wang, Zhao, Yao, Ye & Jiang 2011). This is particularly the case in settings where access to ART is limited (Genberg *et al*. 2009).

### Strategies in confronting stigma

This study provides some insight into the respondents' own acceptance of HIV/AIDS and strategies in confronting stigma. Similar to previous studies, there was evidence that older HIV-positive adults have developed ways of managing life with HIV (Emlet *et al*. 2010; Raveis, Selwyn & Frederickson 2008). However, while many claimed to have largely overcome stigma, there were times and situations where they felt stigmatised because of their HIV status. The state of ‘being or feeling stigmatised’ was not constant. Similarly, perceptions about stigma do not necessarily disappear with time (Dowshen, Binns & Garofalo 2009) Moods fluctuated moving from happiness and hope to worry, sadness and despair over the months depending on the prevailing circumstances. This finding is consistent with previous studies on despondency and stigma (Wright, Zalwango, Seeley, Mugisha & Scholten 2012) where respondents regularly expressed frustration over economic constraints (due to lack of money to meet basic needs), medical problems including those related to HIV and old age, concerns about providing care for children and grandchildren, feelings of sadness and isolation, and lack of support from others as factors associated with stigma. One explanation for the changes in behaviour and opinions about stigma may be: fluctuations in health and support from others, feelings of isolation, food availability, confidence or despondency, the respondents' changing attitudes towards HIV/AIDS as well as a sense of empowerment through intolerance of stigma. Furthermore, fluctuations in opinions and/or perceptions about stigma could be because respondents may be aware of affirmative views of living positively with HIV given through their counselling services.

In this study health-related stigma was present alongside a perception of social stigma. This is probably because health fears referred to by respondents as stigma remain even after a positive diagnosis of HIV. It is worth noting that health fears are at the root of social stigma. Moreover, health fears as an aspect of stigma are broader than the first of four domains of stigma identified by Berger, Ferrans & Lashley (2001). Health itself would ideally be the enduring issue amid increased HIV/AIDS and anti-stigma counselling. However, viewing all HIV/AIDS fears as ‘stigma’ may lead to the self-stigmatisation of reasonable health concerns. Yet these health concerns may not be rooted in stigma alone but are based on testing HIV-positive, having an informed understanding of HIV/AIDS and the availability of HIV treatment. This might explain some of the inconsistencies in respondents' self-perceptions and therefore coping strategies.

### Varying manifestations

The manifestations of HIV-related stigma varied across gender, age, sex and social setting. However, certain manifestations in specific social settings seemingly impacted the psychological wellbeing of respondents more than others. For instance, certain experiences of stigmatisation with respondents' families and in healthcare settings such as fear of being identified as HIV-positive by friends seemed more strongly associated with psychological distress than others. These findings suggest that stigma reduction interventions focusing on influential settings such as health service provision points, and homes, and families may benefit the psychological wellbeing of older HIV-positive persons (Stutterheim, Pryor, Bos, Hoogendijk, Muris & Schaalma 2009).

The study also provided more nuanced insights into the relationships between self-stigma and social stigma as well as the complex effects they have on the wellbeing and quality of life of the respondents. It demonstrated both positive and negative attitude changes associated with HIV-related stigma. The results also show that understanding social norms as well as individual behaviours is useful for informing the development and implementation of effective stigma reduction strategies (Li, Liang, Lin, Wu, Borus & Jane 2010).

The purpose of our study was not to compare levels of stigma between older and younger adults. However, on one hand, similar to other studies, it underscores the substantial presence of HIV stigma (47% had a high stigma score) alongside other forms of stigma (including old age, witchcraft accusations, poverty) in the lives of older persons (AIDS 2001; Anderson 1998; Emlet 2006b; Kuteesa *et al*. 2012; Lavick 1994; Marr 1994). The stigma scores were similar among those with high and low disclosure scores. On the other hand, our study reports relatively high levels of disclosure of HIV-positive status to others (Overall, 55% of respondents had a high disclosure score, meaning they disclosed easily. This finding differs from previous studies that have suggested that older HIV-positive persons have low levels of disclosure (Emlet 2006a; Serovich & Mosack 2003). Nevertheless, it substantiates the theory of stigma and disclosure as a complex social process dependent on several determinants including age and cultural and environmental aspects. It highlights that efforts to alleviate stigma among older persons now or in the future must be context specific.

## Conclusions

HIV- and AIDS-related stigma and discrimination are recognised barriers to effective HIV prevention, diagnosis, treatment care and support. They are associated with poor health and psychosocial outcomes and may lead to greater challenges for this underserved population.

The in-depth analysis in this paper demonstrates the importance of context for understanding the different forms of HIV-related stigma among older people in general and, related to our findings, among those who are living with HIV. The health and psychological wellbeing of many older people in Uganda are intimately affected by the different forms of stigma including old age albeit in different ways and to varying extents.

Because stigma does not have a constant presence, throughout the trajectory of the illness, when caring for HIV-positive older persons, healthcare providers and family members need to be aware of the impact that stigma and discrimination may have on psychosocial wellbeing. Our findings speak to the need for continued education of the general public about HIV/AIDS to prevent the discrimination that older vulnerable adults may face. Furthermore, it is worth noting that it is crucial to include HIV-positive older people in developing interventions (effective prevention and treatment programmes) to address social stigma at community, district and national levels. Their voices need to be heard in order to change society's attitudes about older peoples' sexuality and HIV-positive status.
